# Cytokines in new-onset refractory status epilepticus: ready for clinical use?

**DOI:** 10.1186/s42494-024-00163-6

**Published:** 2024-07-01

**Authors:** Zhenghan Jin, Yixin Zhan, Shijia Chen, Yang Zheng

**Affiliations:** 1 Department of Neurology, The First Affiliated Hospital of Zhejiang Chinese Medical University, Hangzhou, Zhejiang 310000 China; 2 Key Laboratory of Neuropharmacology and Translational Medicine of Zhejiang Province, School of Pharmaceutical Sciences, Zhejiang Chinese Medical University, 54 Youdian Road, Hangzhou, Zhejiang 310006 China

**Keywords:** New-onset refractory status epilepticus, Cytokines, Seizures

## Abstract

New-onset refractory status epilepticus (NORSE) is a rare and challenging condition characterized by refractory status epilepticus in an otherwise healthy patient without obvious causes. Increasing evidence suggests a change in cytokine profiles in NORSE. However, the clinical utility of cytokine testing remains uncertain, primarily because of the lack of robust study designs and limited sample sizes. A recent study published in *Annals of Neurology* investigated the cytokine profiles in both serum and cerebrospinal fluid samples of NORSE patients. The study found elevated levels of CXCL8, CCL2, and MIP-1α in the serum and elevated levels of IL-1ß in the cerebrospinal fluid of NORSE patients compared to those with other forms of refractory status epilepticus (RSE). Furthermore, patients with cryptogenic NORSE had even higher levels of CXCL8, CCL2, and MIP-1α in the serum. Patients with NORSE who exhibited elevated levels of innate immunity cytokines in the serum had worse outcomes at discharge and several months after the NORSE ended. In summary, these findings highlight the association between inflammation-related cytokines and NORSE, providing new insights into clinical diagnosis and treatment approaches.

## Background

New-onset refractory status epilepticus (NORSE) is a unique entity of refractory status epilepticus (SE) that occurs in patients without a prior history of epilepsy and with no readily identifiable causes [1]. NORSE remains a significant clinical challenge due to its poor prognosis and the lack of effective treatments. Currently, biomarkers that can guide treatment strategies and predict disease outcomes are still lacking. There is increasing evidence of elevated levels of pro-inflammatory cytokines, reflecting a hyperactivated innate immune response in patients with NORSE. However, the clinical significance of these cytokine changes in NORSE remains elusive, mainly attributable to the limited sample sizes, the lack of controls, and the absence of standardized methodologies in previous studies. Furthermore, it remains unknown whether the serum or cerebrospinal fluid (CSF) samples should be utilized, as there is little data comparing the cytokines derived from different sources.

## Main text

In a recent issue of *Annals of Neurology*, Hanin et al. investigated the role of cytokines in both the serum and CSF of patients with NORSE [2]. This multicenter study included 98 patients with refractory status epilepticus (RSE), of which 61 were diagnosed with NORSE. Among them, 24 had febrile infection-related epilepsy syndrome (FIRES), a subset of NORSE, which occurs when febrile illness precedes NORSE, and 51 were categorized as cryptogenic NORSE (cNORSE) where the cause remains unknown despite extensive workup. The control groups consisted of patients with autoimmune encephalitis without SE (*n* = 12), patients with pharmacoresistant epilepsy (*n* = 22), and other patients without epilepsy (*n* = 18). Through comprehensive cytokine profiling in both the serum and CSF, this study expands upon existing knowledge regarding the clinical utility of serum and CSF cytokines in the context of NORSE (Table 1). It also provides a foundation for future mechanistic studies and the development of cytokine-targeted treatments for NORSE patients.

**Table 1:** Cytokine alterations in patients with new-onset refractory status epilepticus

	Diagnosis	Findings
**CSF**		
Jun et al. [3]	NORSE	Increased IL-6 levels
Wang et al. [4]	NORSE due to NMDAR encephalitis	Decreased IL-6 level
Hanin et al. (the commented study) [2]	NORSE	Increased IL-6, CXCL8, CCL2, MIP-1α levels
**Serum**		
Jun et al. [3]	NORSE	Increased IL-6 level
Hanin et al. (the commented study) [2]	NORSE	Increased IL-6, TNF-α, CXCL8, MIP-1α, CCL2, IL-12p70, IL-4 and IL-10 levels

*Abbreviations: NORSE* New-onset refractory status epilepticus, *SE *Status epilepticus, *ASMs *Anti-seizure medications, *CSF *Cerebrospinal fluid, *cNORSE *Cryptogenic new-onset refractory status eEpilepticus

 The main findings can be summarized as follows: (1) The cytokine profile changed in both the serum and CSF of patients with SE: The authors compared the cytokine levels in the serum, CSF, and CSF/serum rations between patients with SE and those without SE. A total of eight cytokines in the serum and six in the CSF had significant alterations in patients with SE. (2) Patients with NORSE had a distinct profile of cytokines: Cytokine levels were compared among five subgroups, including NORSE, RSE, and those without SE (pharmacoresistant epilepsy, autoimmune encephalitis, and controls). The elevated level of IL-6, CXCL8, CCL2, and MIP-1α in the serum and the incerased level of IL-1ß in the CSF were observed in NORSE patients. The level of serum CXCL8, CCL2, and MIP-1α was even higher in patients with cNORSE than those with other forms of RSE. (3) Cytokines in the serum were better biomarkers of NORSE compared to the CSF: Serum cytokine disruptions were more pronounced than those in the CSF in NORSE patients. In cNORSE patients, significant increases in cytokine levels were observed exclusively in serum samples. Elevated serum levels of cytokines were more closely linked to the prognosis of NORSE compared to CSF levels.

These findings offer constructive clinical and mechanistic insights. Firstly, in comparison to previous researches [3–10], this study takes a step forward in terms of both sample size and experimental design. The multicenter study, with standardized collection and unbiased analysis of samples, strengthens robust evidence for future reference. Secondly, the study highlights the potential of cytokine levels, especially those in the serum, as biomarkers for disease severity and outcomes in NORSE patients. This outcome is surprising, considering that CSF cytokines have conventionally been perceived as more “reliable” indicators of disease biomarkers. This may be due to the different roles of cytokines in the serum and CSF. In other words, the finding supports the notion that cytokines in the CSF are merely secondary to seizures, while cytokines produced in peripheral inflammation may play a pathogenic role in the development of NORSE. Lastly, the study establishes a link between cytokines levels and the subtype of SE, ictal burdens, and outcomes at discharge and follow-up, providing evidence for the pathogenic role of cytokines in NORSE. The positive correlations observed, especially with IL-6, CXCL8, MIP-1a, and CCL2, highlight the crucial involvement of specific innate immune pathways in disease pathogenesis. These findings suggest potential avenues for clinical implementation of anti-inflammatory interventions, such as IL-6 blockers like tocilizumab, and anti-CXCL8 therapy such as reparixin, in the management of NORSE.

When interpreting the results, it is also crucial to consider several points in mind. To begin with, the level of cytokines may fluctuate throughout the course of the disease. In this study, there was a delay in sample collection among patients with NORSE, with a median interval of 11 days for serum samples and 8 days for CSF samples. Although the authors have revealed a lack of association between cytokine levels and the time of delay, it is possible that certain cytokines that transiently arise in the early phase of the disease or rapidly decrease after treatment may have been missed. Therefore, repeated measurements may be necessary to fully capture the dynamic progression of the disease. Moreover, CSF samples were selectively collected in certain patients based on the discretion of the treating physician. This introduces potential bias, as lumbar punctures may be conducted primarily in cases with a more refractory course or evident signs of inflammatory or autoimmune origins. Prospective studies incorporating paired serum and CSF samples are needed to validate those results.

Despite the insights gained from the study, there are still challenges to address before the clinical use of cytokines in NORSE patients. Firstly, concerns regarding the specificity of cytokines can be better addressed by the appropriate selection of controls. The authors established a comprehensive set of controls, including autoimmune encephalitis to represent acute neuroinflammatory conditions and pharmacoresistant epilepsy to represent acute seizures, along with a non-epileptic baseline for comparison. However, incorporating acute non-neurological inflammatory controls would clarify whether the increase in peripheral inflammatory factors in SE patients is attributable to NORSE or concurrent peripheral inflammation. Secondly, the threshold of cytokines varies based on the specimen, methods of testing, and the demographic profiles of patients. This study used normative thresholds from its control patients, which may not be universally applicable to other centers. Setting the right threshold values requires collaborative efforts across multiple centers, and is essential for the individualized application of cytokines in patients with NORSE.

## Conclusions

Overall, this study, with a relatively large sample size and standardized methodologies, highlights the utility of cytokine disturbances in NORSE. These findings expand the previous knowledge on NORSE and innate immunity by comparing serum and CSF samples and by incorporating various clinical outcomes. The results suggest certain cytokines could serve as prognostic and treatment biomarkers in NORSE, which may also be potential targets for future precision therapies.

## Data Availability

Not applicable.

## Abbreviations

ASMs: Anti-seizure medications
CSF: Cerebrospinal fluid
cNORSE: Cryptogenic new-onset refractory status epilepticus
NORSE: New-onset refractory status epilepticus
RSE: Refractory status epilepticus
SE: Status epilepticus
